# Endoscopy-assisted anterior cervical discectomy and fusion with internal fixation vs conventional surgery in the treatment of cervical disc herniation

**DOI:** 10.20452/wiitm.2024.17888

**Published:** 2024-07-31

**Authors:** Haicun Zhang, Yanbo Lin, Canglu Wu, Fangling Cheng, Danqing Bao, Yuyan Chen

**Affiliations:** Department of Spinal Surgery, Zhoushan Dinghai Guanghua Hospital, Zhoushan, Zhejiang Province, China

**Keywords:** anterior cervical spine operation, cervical disc herniation, cervical vertebra, endoscope, fusion

## Abstract

**INTRODUCTION:**

Cervical disc herniation (CDH) is a common condition, usually caused by excessive strain or trauma to the spine. Initially, it is treated conservatively; however, complex and resistant cases may require a surgical intervention.

**AIM:**

We aimed to compare the clinical effect of endoscopy-assisted anterior cervical discectomy and fusion (ACDF) with internal fixation and conventional surgery in the treatment of CDH.

**MATERIALS AND METHODS:**

Patients with CDH who underwent ACDF with fixation at the Zhoushan Dinghai Guanghua Hospital were enrolled. Of them, 10 individuals were treated with conventional ACDF (conventional surgery group), and the other 10 with endoscopy-assisted ACDF (endoscopy-assisted surgery group). The general characteristics, postoperative Japanese Orthopedic Association (JOA), visual analogue scale (VAS), 12-Item Short Form Survey Physical Component Summary (SF-12 PCS), and SF-12 Mental Component Summary (SF-12 MCS) scores, physiological stress response, rate of the improved JOA score (RIS), hemoglobin level, and bone graft fusion were compared between the groups.

**RESULT:**

Outcomes of the patients treated with endoscopy-assisted surgery were clearly superior to those observed in the conventional surgery group. The postoperative JOA, VAS, SF-12 PCS, and SF-12 MCS scores and RIS in the endoscopy-assisted surgery group were higher than in the conventional surgery group (*P *<⁠0.05). Following operation, there were significant differences between the 2 groups with respect to RIS at 1 week and 6 months postsurgery and hemoglobin levels on postoperative day 2. Changes in heart rate and diastolic blood pressure in the endoscopy-assisted surgery group were less pronounced than in the conventional surgery group (*P *<⁠0.05), and the fusion rate was significantly higher in the former group (90% vs 80%, respectively).

**CONCLUSION:**

Endoscopy-assisted ACDF with internal fixation has a greater clinical therapeutic effect than the conventional approach in the treatment of CDH. It is associated with a higher bone graft fusion rate and reduced intraoperative blood loss.

## INTRODUCTION 

Cervical disc herniation (CDH) is a common spinal disorder. It is usually caused by cervical disc degeneration, which leads to formation of osteophytes at the posterior edge of the vertebral body. A herniated disc compresses the nerve roots, spinal cord, and vertebral artery, causing a variety of adverse symptoms.^1^ In recent years, due to lifestyle changes and increasing prevalence of sedentary work, CDH has been observed in younger people, and the number of individuals suffering from chronic cervical vertebral injury and degeneration has been on the rise. CDH may also be caused by trauma, for example, during a traffic accident.^2^^;^^3^ In the case of symptom aggravation despite conservative treatment, surgery should be performed as soon as possible.

With the rapid development of spine endoscopy, this approach has been successfully applied in the field of traditional open surgery; thus, treatment of patients with spinal conditions has gradually entered a new era.^4^^;^^5^ Anterior cervical spine discectomy and fusion (ACDF) is a gold‑standard surgical treatment for cervical spine diseases, with proven clinical efficacy.^6^ As compared with posterior surgery, anterior surgery can directly relieve compression at the front of the spinal cord by removing factors causing the compression, such as degenerative disc tissue, osteophytes at the posterior margin of the vertebral body, hypertrophic or ossified posterior longitudinal ligament, and hyperplasia of the uncinate joint. Intervertebral bone graft can effectively restore the height of the intervertebral space and stability of the diseased segment, and help maintain physiological curvature of the cervical spine. ACDF alleviates pain and other symptoms by reducing neural compression, and contributes to the recovery of function lost due to the compression. Fusion procedures facilitate restoration and maintenance of cervical spine stability. ^7^

ACDF with internal fixation can ensure immediate stability of the cervical spine, while intervertebral fusion can reduce the occurrence of cervical kyphosis at the later stage. The method of internal fixation depends on the case.^8^^;^^9^ However, due to anatomical limitations, the surgical field of view within the intervertebral space is often small during anterior cervical spine surgery, which makes the procedure more difficult and potentially affecting hemostasis. Solutions have been sought to overcome this problem.^10^^;^^11^ Owing to continuous advancements in endoscopic technology, improvement of endoscopic instruments, and enhancement of surgical skills among physicians, endoscopic intervertebral disc surgery has become widely used in the treatment of cervical spine diseases, as it allows for complete and direct vision of the surgical field under endoscopy guidance.^12^^;^^13^ During endoscopy‑assisted anterior cervical discectomy, the operating field is clearly exposed, which enables the surgeon to accurately treat the lesion site.^14^ Endoscopy‑assisted procedures are associated with fewer surgical incisions, faster postoperative recovery, and a lower rate of complications, as compared with open surgery.^15^

We aimed to explore the surgical effect of endoscopy‑assisted ACDF and internal fixation, accumulate relevant surgical experience, discuss the characteristics of the procedure and the related surgical difficulties, and gather data allowing for further development of minimally invasive anterior cervical spine surgery.

## AIM 

The aim of this study was to compare the clinical outcomes of endoscopic‑assisted ACDF with internal fixation vs traditional fixation methods and to evaluate its role in the surgical treatment of CDH.

## MATERIALS AND METHODS 

### Patients 

A total of 20 patients (12 men, 8 women) admitted to the Zhoushan Dinghai Guanghua Hospital between January 2023 and December 2024 for ACDF and internal fixation were included. The first 10 consecutive patients were treated with conventional surgery, while the other 10 underwent endoscopy‑assisted ACDF. Mean (SD) age of the patients was 51.6 (6.3) years (range, 42–66 years). All operations were performed by the same group of doctors with equal qualifications. Data were collected and analyzed using a double‑blind method. All patients accepted the study regulations and signed an informed consent to participate. The study protocol was approved by the ethics committee of the Zhoushan Dinghai Guanghua Hospital (ZDGH318).

The inclusion criteria comprised: 1) long‑term treatment at the Zhoushan Dinghai Guanghua Hospital; 2) clinical condition requiring surgical intervention and eligibility for surgery; 3) single‑level radiculopathy or single‑segment cervical spondylotic myelopathy; 4) no mental disorders; 5) a lack of response to conservative treatment for more than 3 months; 6) consent for participation.

The exclusion criteria were as follows: 1) refusal to participate in the study; 2) a history of cervical spine surgery for cervical spondylosis; 3) multisegmental disc disease; 4) use of anterior cervical plate for fixation; 5) use of subtotal resection; 6) osteoporosis and poor cervical stability complicated with inflammation.

### Treatment methods 

#### Endoscopy‑assisted surgery 

The procedure was performed under general anesthesia, with the patient placed in the conventional supine position. Before the surgery, the incision location was determined by a C‑arm X‑ray device (Cios Alpha, Siemens Healthineers, Erlagen, Germany). A small medial incision was made in the front, exposing the intervertebral space along the anterior cervical spine, and an automatic small retractor was placed and fixed. Following stretching, the intervertebral space was cut, and most of the nucleus pulposus was stripped under direct vision to reach the posterior edge of the intervertebral disc. The posterior edge of the intervertebral disc was monitored by endoscopy. The posterior edge of the nucleus pulposus was cleaned, proliferating osteophytes and the posterior longitudinal ligament were removed, and the nerve roots and spinal cord were exposed. Subsequently, the endoscopic system was removed, a Zero‑P fusion device was implanted in the intervertebral space, and conventional fluoroscopy was performed. The procedure was finished with wound irrigation and suturing.

#### Conventional surgery 

The preprocedural protocol and initial stages of the conventional surgery were the same as described above for the endoscopy‑assisted procedure. The same approach was used to access the cervical spine. Following stretching, the vertebral space was cut, the nucleus pulposus was removed under direct vision, the posterior edge of the nucleus pulposus was cleaned, hyperplastic osteopathy and posterior longitudinal ligament were removed, and the nerve roots and spinal cord were exposed. The Zero‑P fusion apparatus was implanted in the intervertebral space, and conventional fluoroscopy was followed by irrigation and suturing.

**Table 1:** Baseline patient characteristics

Parameter		Conventional surgery group (n = 10)	Endoscopy‑assisted surgery group (n = 10)	t/χ2	*P *value
Sex, n (%)	Men	7 (70)	5 (50)	0.32	0.3
	Women	3 (30)	5 (50)		
Age, mean (SD)	53.4 (7.41)	50.8 (6.39)	4.87	0.22	
Body mass index, kg/m2, mean (SD)	26.3 (4.12)	27.8 (5.05)	3.22	0.33	
Smoking, n (%)	6 (60)	5 (50)	0.16	0.19	
Excessive alcohol consumption, n (%)	2 (20)	1 (10)	0.43	0.19	
Comorbidities, n (%)					
Diabetes mellitus	3 (30)	5 (50)	0.27	0.62	
Hypertension	4 (40)	3 (30)	7.07	0.73	
Coronary heart disease	3 (30)	2 (20)	6.32	0.53	
Hyperlipidemia	4 (40)	3 (30)	0.12	0.32	

Standard imaging (magnetic resonance imaging [MRI] and computed tomography [CT]) of the cervical spine in anteroposterior and lateral views was performed in all patients before the surgery for assessment and definitive diagnosis, and follow-up X‑ray, CT, and MRI were carried out postoperatively. Both groups were operated on by the same associate chief physician specializing in spine surgery. Postoperative drainage tube was routinely placed in both groups. On postoperative day 1, all patients were mobilized while wearing a 2‑piece neck brace for support. Blood tests were performed on the second day postsurgery, and changes in hemoglobin level were recorded. Decompression was monitored by MRI within the first postoperative week, and the visual analogue scale (VAS) score was evaluated on the second day and in the first week after surgery.

### Observation indicators 

General baseline characteristics (age, sex, medical history) were recorded. Data on smoking, alcohol consumption, and underlying diseases (internal diseases, osteoporosis, diabetes, hypertension, coronary heart disease, hyperlipidemia) were collected and compared between the groups. Intraoperative complications, such as tolerance to anesthesia, cerebrospinal fluid leakage, and nerve or blood vessel injury, were analyzed. Parameters related to treatment efficacy were also assessed, including operation time, changes in hemoglobin level, pain scores on the second day postsurgery, as well as postoperative decompression and fusion. Postoperative anteroposterior and lateral X‑rays were performed regularly to monitor changes in height of the cervical vertebra and bone graft fusion status. The 12‑item Short Form Survey (SF‑12) Physical Component Summary (PCS) was employed to evaluate the physical health of the patients,including physical pain, physiological intelligence, physiological function, and general health. Mental health was assessed using the SF‑12 Mental Component Summary (MCS), which evaluates aspects such as vitality, mental health, social functioning, and emotional functioning. The level of pain experienced by the patients was evaluated according to a 10‑point VAS (with 0 indicating no pain, and 10 indicating the worst pain possible). 

The patients were followed by means of telephone calls or outpatient visits. The follow‑up period was 6 months.

Pre- and postoperative scores were calculated according to the Japanese Orthopedic Association (JOA) criteria.^16^ Neurological function improvement after vs before surgery was calculated using the following formula: JOA improvement rate (RIS) = (postoperative score – preoperative score)/(17 – preoperative score) × 100%. RIS greater than 75% was considered excellent, 50%–75% was considered good, 25%–49% was deemed medium, and 0%–24% or a JOA score lower than before surgery was considered poor.

### Statistical analysis 

The SPSS 25.0 software (IBM Corp., Armonk, New York, United States) was employed for statistical analysis. Study data are presented as numbers and percentages, and were compared using the χ2 test. Quantitative data are shown as mean (SD), and the t test was used for their comparison. A P value below 0.05 was considered significant.

### RESULTS 

### General characteristics 

As shown in Table 1 , there were no significant differences between the 2 groups with respect to sex, age, body mass index, smoking status, alcohol consumption, or underlying diseases.

### Clinical outcomes

**Figure 1:**
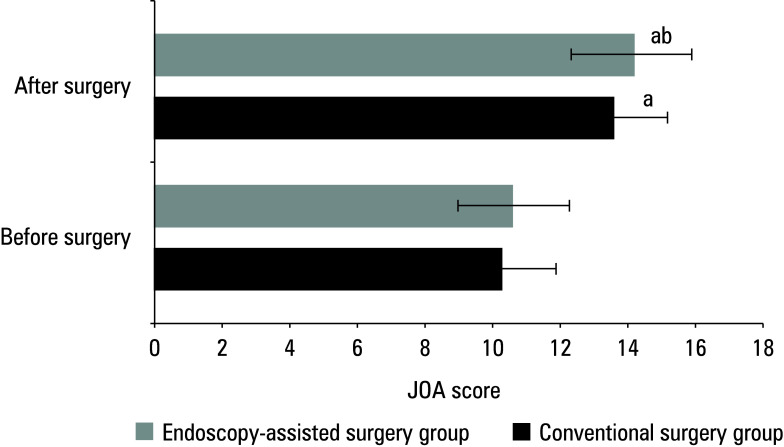
Differences in the Japanese Orthopedic Association (JOA) score between the groups

In the conventional surgery group, the mean (SD) pre‑ and postoperative JOA scores were 10.21 (0.86) and 13.54 (0.93), respectively, while in the endoscopy‑assisted surgery group, the scores were 10.74 (0.86) and 14.13 (0.86), respectively. The preoperative JOA score was similar between the groups (P >0.05). Following surgery, the score improved in both groups; however, the difference was more pronounced in the group treated with endoscopy‑assisted surgery (P <0.05)Figure 1.

a P <0.05 vs values before surgery in the same group

b P <0.05 vs conventional surgery group

**Figure 2:**
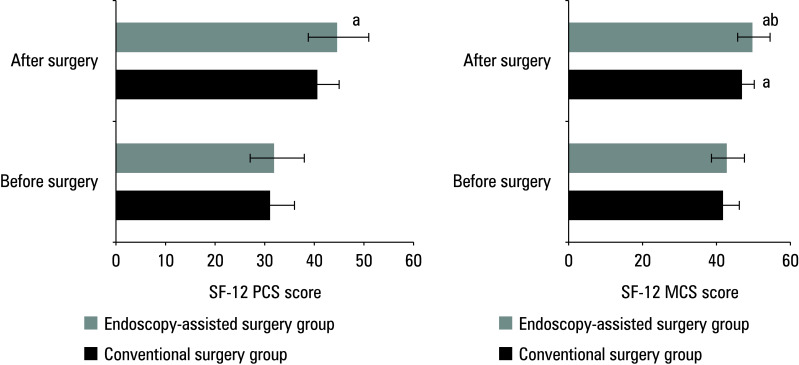
Difference in 12‑item Short Form Survey (SF ‑12) Physical Component Summary (PCS) and Mental Component Summary (MCS) scores between the groups

a P <0.05 vs values before surgery in the same group

b P <0.05 vs conventional surgery group

### Physical and mental well‑being 

As shown in A, the mean (SD) SF‑12 PCS scores in the conventional surgery group were 31.71 (8.85) and 40.47 (11.32) before and after the operation, respectively. In the endoscopy‑assisted surgery group, the respective scores were 32.62 (9.73) and 45.41 (10.34). Before surgery, the SF‑12 PCS scores were similar between the groups, while the postoperative scores differed significantly. In both groups, physical well‑being improved postsurgery, as reflected by higher SF‑12 PCS scores. With respect to mental well‑being, surgery resulted in a significant improvement in this domain in both groups. The mean (SD) pre‑ and postoperative SF‑12 MCS scores were 42.63 (11.53) and 47.47 (11.32), respectively, in the conventional surgery group, and 43.38 (12.07) and 50.73 (11.41) in the endoscopy‑assisted surgery group. The postoperative scores differed significantly between the groups Figure 2B.

### Postoperative visual analogue scale scores 

**Figure 3:**
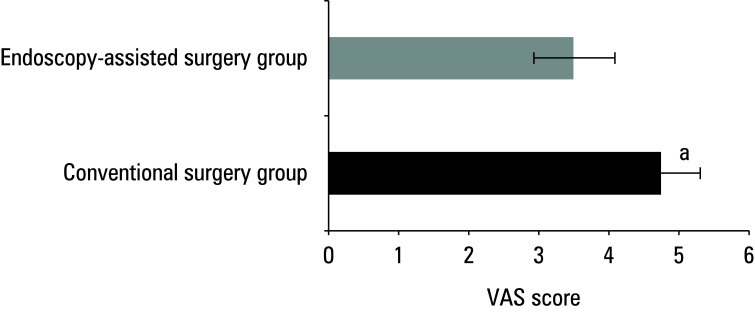
Difference in postoperative visual analogue scale (VAS) scores between the groups

The mean (SD) VAS score on the second day after surgery in the conventional surgery group was 4.68 (1.53), while in the patients treated with endoscopy‑assisted surgery, it was 3.52 (1.36)(P <0.05) Figure 3.

### Rate of improved Japanese Orthopedic Association score 

**Figure 4:**
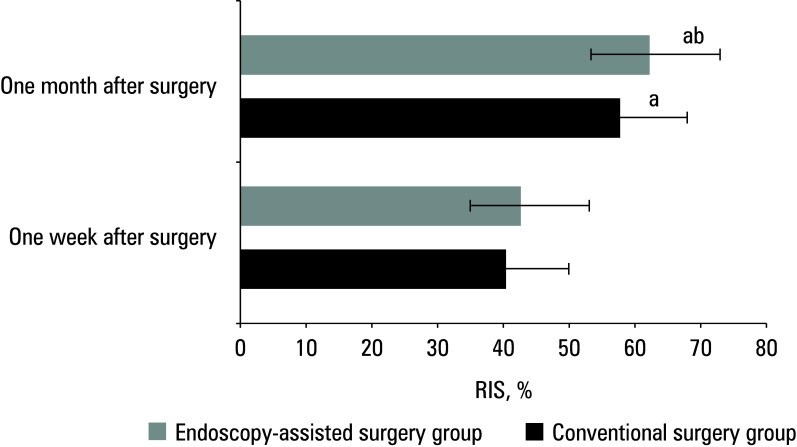
Difference in the rate of improved Japanese Orthopedic Association score (RIS)

The mean (SD) RIS in the conventional surgery group was 40.63% (5.32%) at postoperative week 1 and 57.31% (4.91%) at postoperative month 6 (P <0.05). In the endoscopy‑assisted surgery group, the respective values were 43.38% (6.07%) and 63.35% (6.13%) (P <0.05). The difference between the groups was significant at the latter time point Figure 4.

### Physiological stress response 

The mean (SD) heart rate (HR) in the conventional surgery group was 79.39 (10.53) bpm and 92.79 (12.25) bpm before and 1 week after surgery, respectively. In the endoscopy‑assisted surgery group, the respective values were 79.83 (11.37) bpm and 88.62 (10.53) bpm. 

a P <0.05 vs endoscopy‑assisted surgery group

a P <0.05 vs value 1 week postsurgery in the same group

b P <0.05 vs conventional surgery group

**Figure 5:**
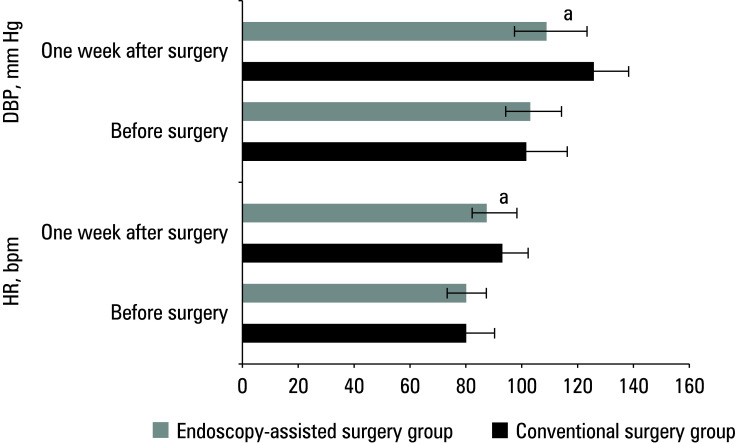
Difference in markers of physiological stress response between the groups

a P <0.05 vs conventional surgery group

Abbreviations: DBP, diastolic blood pressure; HR, heart rate

In the conventional surgery group, the mean diastolic blood pressure (DBP) before surgery vs 1 week after was 102.61 (7.34) mm Hg vs 126.59 (8.05) mm Hg. The respective values in the endoscopy‑assisted surgery group were 103.83 (5.72) mm Hg. and 112.23 (6.72) mm Hg.

With respect to both parameters, changes in the endoscopy‑assisted surgery group were significantly less pronounced than in the patients treated with conventional surgery Figure 5.

### Hemoglobin levels 

**Figure 6:**
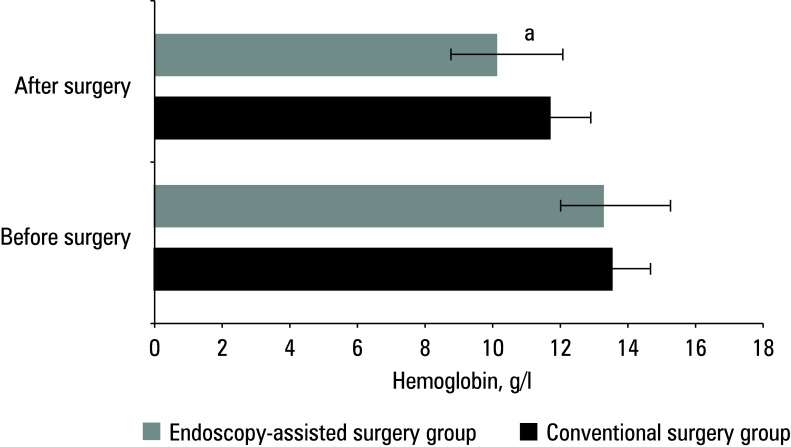
Difference in hemoglobin levels between the groups

In the conventional surgery group, the mean (SD) hemoglobin level was 13.7 (1.45) g/l preoperation and 11.8 (1.06) g/l on postoperative day 2. In the patients treated with endoscopy‑assisted surgery, the respective values were 13.6 (0.7) g/l and 10.3 (1.32) g/l. The post‑ operative hemoglobin level differed significantly between the groups Figure 6.

### Bone graft fusion 

**Figure 7:**
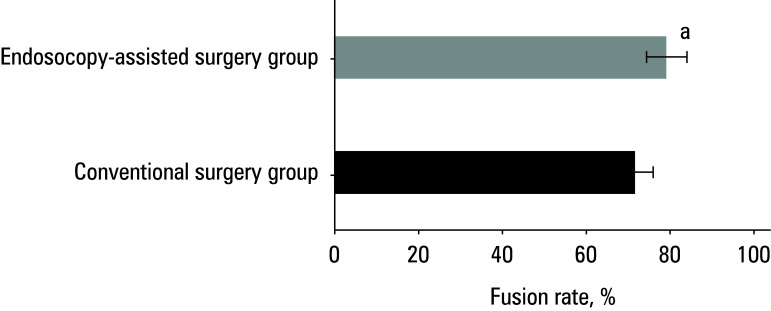
Difference in graft fusion rate between the groups

X‑rays of the cervical spine performed on the first day postoperation and at the last follow‑up visit suggested that position of the internal fixation device was satisfactory in both groups, without fracture, collapse, or loosening. In the conventional surgery group, there were 5 cases of interbody fusion, 3 cases of suspicious fusion, and 2 cases of no fusion at the last follow‑up, with a fusion rate of 80%. In the endoscopy‑assisted surgery group, there were 6 cases of interbody fusion, 3 cases of suspicious fusion, and 1 case of no fusion, yielding a fusion rate of 90%. The difference between the groups was significant Figure 7.

#### Intra‑ and postoperative complications 

As shown in Table 2 , there were significant differences in postoperative drainage volume, intraoperative blood loss, operation time, and incision length between the 2 groups.

## DISCUSSION 

Along with unfavorable changes in people’s lifestyles, the incidence of cervical spondylosis is gradually increasing. Degenerative lesions in the cervical intervertebral discs can lead to cervical disc protrusion, ossification of the posterior longitudinal ligament, facet joint hypertrophy, and osteophyte formation on the anterior and posterior margins of the vertebral bodies, all of which can cause compression of the spinal cord nerves and corresponding neurological dysfunction. ^17^^;^^18^ Percutaneous endoscopic spine surgery has been associated with successful outcomes in clinical practice, and the development of endoscopic techniques has transformed the field of spinal surgery.^19^ Zero‑P internal fixation represents an organic combination of minimally invasive orthopedic techniques and ACDF. Zhang et al^20^ demonstrated that the use of Zero‑P internal fixation for ACDF is superior to traditional anterior cervical fixation. However, as shown by Huang et al,^21^ changes in post‑ vs preoperative C2–7 angle significantly influence the occurrence of swallowing difficulty in patients undergoing implantation of the Zero‑P system for intervertebral fusion surgery. Zhang et al^22^ proposed that ACDF is an ideal choice for single‑level cervical spine surgery, as it effectively reduces the incidence of dysphagia and degenerative changes in patients. This study compared clinical outcomes of endoscopy‑assisted ACDF with internal fixation vs conventional ACDF for the treatment of cervical disc protrusion. We observed that endoscopy‑assisted ACDF with internal fixation shows significant advantages over conventional surgery in terms of clinical effectiveness. Postoperative pain relief, functional improvement, and physiological indicators were superior in the endoscopy‑assisted surgery group, as compared with the conventional surgery group, with higher rates of postoperative bone graft fusion and less intraoperative blood loss. There fore, endoscopy‑assisted surgery may be a more effective option in the treatment of cervical disc protrusion.

a P <0.05 vs conventional surgery group

a P <0.05 vs conventional surgery group

ACDF is considered an effective surgical method for treating cervical spondylosis, capable of alleviating pain, improving daily function, and enhancing patient quality of life.^23^^;^^24^^;^^25^ Prablek et al^26^ demonstrated significant efficacy of ACDF in treating myelopathic cervical spondylosis, which was further confirmed in studies by Li et al^27^ and Cao et al.^28^ These findings are consistent with our study, which also indicated excellent patient outcomes in the endoscopy‑assisted surgery group. Postoperatively, the patients treated with endoscopy‑assisted surgery demonstrated significantly higher improvements in JOA, VAS, SF‑12 PCS, and SF‑12 MCS scores, and exhibited greater RIS, as compared with the conventional surgery group. This suggests that during endoscopy‑assisted surgery, the lesion location can be identified more precisely and accurately, allowing for more thorough removal of disc protrusions and alleviation of nerve compression. This translates into greater postoperative symptom relief and functional improvement. Patients treated with endoscopy‑assisted surgery may experience faster recovery due to reduced surgical trauma, allowing for earlier initiation of rehabilitation training and activity resumption, which aids in accelerating symptom relief and functional recovery. Endoscopy‑assisted surgery potentially reduces surgical trauma and tissue damage, lowering the incidence of postoperative complications and enabling patients to return to normal life and resume work activities more quickly.

In this study, the patients treated with endoscopy‑assisted surgery exhibited significantly better RIS (improvement in JOA scores) at postoperative week 1 and month 6, as well as lower hemoglobin levels on the second postoperative day, as compared with the conventional surgery group. This indicates that in the endoscopy‑assisted surgery group, postoperative recovery of neurological function and physical strength was faster, with less decline in hemoglobin levels. This is potentially related to the finer surgical technique, reduced trauma, and fewer postoperative complications in this group. Changes in HR and DBP were less pronounced in the endoscopy‑assisted surgery group than in the conventional surgery group, suggesting a reduced physiological stress response postoperatively. Endoscopy‑assisted surgery may decrease surgical trauma and postoperative inflammatory responses, thereby alleviating physiological stress reactions. The endoscopy‑assisted surgery group also exhibited a significantly higher bone graft fusion rate and less intraoperative blood loss than the conventional surgery group. This suggests that endoscopy‑assisted surgery can better preserve the integrity of bone tissue, promote bone healing, reduce intraoperative blood loss, and further mitigate surgical risks. In summary, the findings of this study once again emphasize the advantages of endoscopy‑assisted surgery in the treatment of cervical disc protrusion, including rapid postoperative recovery, fewer postoperative complications, improved neurological function recovery, and higher rates of bone graft fusion. These advantages render endoscopy‑assisted surgery the preferred method for the treatment of cervical disc protrusion.

Of note, performing cervical spondylosis surgery requires advanced surgical skills. Endoscope is a complex piece of equipment; therefore, during its application, it is necessary to strictly follow the manufacturer’s instructions to avoid contamination during the operation.

Limitations of the current study comprise a retrospective design and a small sample.

## CONCLUSIONS 

Endoscopy‑assisted ACDF with internal fixation is associated with better clinical therapeutic outcomes and bone graft fusion rate, and lower intraoperative blood loss than conventional surgery. It offers higher safety and effective nerve decompression. Our results further support the effectiveness and benefits of endoscopy‑assisted surgery in the treatment of cervical disc protrusion, providing patients with better treatment options.

**Table 2:** Comparison of intra‑ and postoperative complications between the groups

Parameter	Conventional surgery group (n = 10)	Endoscopy‑assisted surgery group (n = 10)	t/χ2	*P *value
Wound infection rate, n (%)	0	0	0	–
Nerve root injury, n (%)	0	0	0	–
Mild postoperative dysphagia, n (%)	2 (20)	0	0.76	0.61
Dural injury, n (%)	3 (30)	1 (10)	0.85	0.13
Postoperative drainage volume, ml, mean (SD)	32.64 (9.16)	28.76 (10.73)	0.76	0.03
Intraoperative blood loss, ml, mean (SD)	79.53 (10.81)	63.68 (9.72)	3.67	0.004
Operation time, min, mean (SD)	1.59 (0.39)	1.18 (0.85)	2.08	0.01
Incision length, cm, mean (SD)	4.03 (0.45)	4.32 (82)	3.08	0.03

P values <0.05 were considered significant.
